# Cardiac Drugs in ACHD Cardiovascular Medicine

**DOI:** 10.3390/jcdd10050190

**Published:** 2023-04-24

**Authors:** Magalie Ladouceur, Estibaliz Valdeolmillos, Clément Karsenty, Sébastien Hascoet, Pamela Moceri, Laurianne Le Gloan

**Affiliations:** 1 Adult Congenital Heart Disease Medico-Surgical Unit, European Georges Pompidou Hospital, 75015 Paris, France; 2 Centre de Recherche Cardiovasculaire de Paris, INSERM U970, Université de Paris Cité, 75015 Paris, France; 3 Marie-Lannelongue Hospital, Paediatric and Congenital Cardiac Surgery Department, Centre de Référence des Malformations Cardiaques Congénitales Complexes M3C Groupe Hospitalier Saint-Joseph, Paris-Saclay University, 92350 Le Plessis Robinson, France; 4 UMRS 999, INSERM, Marie-Lannelongue Hospital, Paris-Saclay University, 92350 Le Plessis Robinson, France; 5 Pediatric and Congenital Cardiology, Children’s Hospital CHU Toulouse, 31300 Toulouse, France; 6 Institut des Maladies Métaboliques et Cardiovasculaires, Université de Toulouse, Institut National de la Santé et de la Recherche Médicale (INSERM), U1048, 31300 Toulouse, France; 7 UR2CA, Equipe CARRES, Faculté de Médecine, Université Côte d’Azur, 06000 Nice, France; 8 Cardiologie Congénitale Adulte, Institut du Thorax, CHU de Nantes, 44000 Nantes, France

**Keywords:** heart failure, trials, drug therapy, pharmacological therapy, adult congenital heart disease

## Abstract

Adult congenital heart disease (ACHD) is a growing population that requires life-long care due to advances in pediatric care and surgical or catheter procedures. Despite this, drug therapy in ACHD remains largely empiric due to the lack of clinical data, and formalized guidelines on drug therapy are currently lacking. The aging ACHD population has led to an increase in late cardiovascular complications such as heart failure, arrhythmias, and pulmonary hypertension. Pharmacotherapy, with few exceptions, in ACHD is largely supportive, whereas significant structural abnormalities usually require interventional, surgical, or percutaneous treatment. Recent advances in ACHD have prolonged survival for these patients, but further research is needed to determine the most effective treatment options for these patients. A better understanding of the use of cardiac drugs in ACHD patients could lead to improved treatment outcomes and a better quality of life for these patients. This review aims to provide an overview of the current status of cardiac drugs in ACHD cardiovascular medicine, including the rationale, limited current evidence, and knowledge gaps in this growing area.

## 1. Introduction

Major advances in the management of children with congenital heart disease (CHD) have evolved over the past six decades. As a result, most of the children reach adulthood, and the population of adults with CHD is exponentially growing. This evolution is particularly marked for patients with complex CHD. Consequently, the spectrum of congenital lesions is changing over time, with more patients having complex CHD reaching older age with additional acquired comorbidities.

Pharmacotherapy in adult CHD (ACHD) is largely supportive and is used to manage complications such as arrhythmias, heart failure (HF), and pulmonary hypertension in addition to interventional or surgical procedures.

Treatment for cardiovascular disease aims to reduce morbidity and mortality and to improve patient quality of life. The properly designed randomized controlled trial is recognized as providing the highest level of evidence in determining guidelines for therapeutic practice [1]. However, the evidence for such treatment in CHD is scarce. Drug therapy in ACHD is based on limited clinical data and remains mostly empirical. Therapy in CHD, such as those with a systemic right ventricle (RV) dysfunction, tetralogy of Fallot or Fontan circulation failed to demonstrate any conclusive effect, mainly because the methodological quality of studies was low, due to lack of a randomized and controlled design, small sample size, and incomplete follow-up. Indeed, the rare nature and the heterogeneity of CHD and a relatively low short-term incidence of major adverse cardiac events significantly limits the power that can be achieved by prospective trials with relatively limited resources. Therefore, there are no formal guidelines on drug therapy for ACHD, making recommendations challenging. This review examines the growing ACHD area of drug therapy, discussing its rationale, limited current evidence, and knowledge gaps.

## 2. Cardiac Drugs in ACHD-Related Heart Failure 

### 2.1. Subaortic Left Ventricle Failure

In conjunction with surgical or interventional repair of residual anatomic lesions, medical treatment is always proposed to ACHD patients with HF.

HF with reduced ejection fraction (HFrEF) of the morphologic left ventricle (LV) is defined as an ejection fraction (EF) of 40% or less [2], and this seems to be also applicable to ACHD. ACHD patients have typically been excluded from adult HF clinical trials, and there are no trials with hard clinical endpoints pertaining to ACHD patients. Therefore, international guidelines suggest that diuretics, angiotensin-converting enzyme inhibitors, angiotensin receptor blockers, angiotensin receptor/neprilysin inhibitors, mineralocorticoid receptor antagonists, βblockers, and now sodium-glucose cotransporter-2 inhibitors should be used in this population [2]. There is theoretical evidence to support the use of angiotensin converting enzyme inhibitors and, if not tolerated, angiotensin receptor blockers in the treatment of asymptomatic or symptomatic HF ACHD patients. Similarly, the evidence for using β-blockers, such as carvedilol, metoprolol, bisoprolol, and nebivolol, may also be extrapolated to the ACHD population. In a large retrospective study of 4358 adults with CHD and a systemic LV, 12% had a left ventricular systolic dysfunction; the prevalence was higher in right-sided compared with left-sided lesions [3]. In this study, 56% received guideline-directed medical therapy, resulting in a 6% increase in left ventricle ejection fraction (LVEF).

HF with preserved ejection fraction (HFpEF) is less common in ACHD patients, but may be underdiagnosed [4]. Diuretics are used for symptom relief, β-blockers may help by prolonging ventricular filling. Recent large, randomized control trials have shown that sodium–glucose cotransporter-2 inhibitors (SGLT2i) improve outcomes in HFpEF [5,6]. This was an important finding, knowing up to now, the treatment of HFpEF has been characterized by lack of therapies that improve prognosis. SGLT2i are theoretically promising in ACHD with reduced and preserved LVEF but need further investigations.

The APPROPRIATE study (ACE inhibitors for Potential PRevention Of the deleterious effects of Pulmonary Regurgitation In Adults with repaired TEtralogy of Fallot) was a randomized double-blinded, placebo-controlled trial comparing ramipril 10 mg daily with placebo in 64 patients with repaired tetralogy of Fallot and at least moderate pulmonary regurgitation [7]. Post-hoc echocardiographic analysis revealed that six months of treatment appear to stabilize systolic and diastolic LV function.

### 2.2. Sub-Pulmonary Right Ventricle Failure

Scarce data are available considering sub-pulmonary right ventricular (RV) HF. It can be the result of either pressure overload or volume overload. When present, residual anatomic lesions must be addressed by surgery or interventional approach.

In the case of pressure overload secondary to pulmonary arterial hypertension (PAH), ultimately Eisenmenger syndrome, medical therapy focuses on the pulmonary circulation with introduction of PAH-specific drugs, namely endothelin-receptor antagonists, phosphodiesterase-5 inhibitors, and prostacyclin analogues [8] (see Section 3). Risk assessment is therefore recommended for all patients, and initial oral combination or sequential combination therapy must be proposed.

In the case of HF due to volume overload, mainly represented by patients with severe pulmonary regurgitation in repaired tetralogy of Fallot, diuretics can be used to relieve symptoms. In asymptomatic patients, no medications are indicated.

The REDEFINE trial (Right Ventricular Dysfunction in Tetralogy of Fallot: Inhibition of the Renin-Angiotensin-Aldosterone System) is a multicenter, prospective, randomized, double-blind, placebo-controlled study [9]. Ninety-five adults with repaired tetralogy of Fallot and mild RV dysfunction in the absence of severe valvular lesions, were enrolled. Forty-seven received 150 mg losartan daily and forty-eight received placebo. Patients were treated for 21 ± 4 months. Losartan did not significantly improve RV ejection fraction (RVEF) in comparison with placebo. It also did not improve peak aerobic exercise capacity. Favorable effects of losartan in symptomatic patients were not statistically significant. Losartan should not be prescribed routinely in these patients to prevent the progression of RV dysfunction and RVHF.

### 2.3. Systemic Right Ventricle Failure

ACHD with a systemic right ventricle (sRV) cannot support cardiac output in the long run. Tricuspid regurgitation, impaired coronary flow reserve, myocardial fibrosis, and ventricular dyssynchrony are highly interconnected and contribute to sRV failure. Clinical HF is common in patients with sRV, and over time [10], the HF burden in patients with atrial switch repair and congenitally corrected transposition of the great arteries will only increase. Recent improvements in the management of HF may improve sRV failure management.

Beta blockers are considered as central in HF management, although results in sRV are conflicted. Bisoprolol and carvedilol, in small cohorts of patients, were safe and tended to improve RV/LV volumes and function [11,12,13]. RV dilatation has been related to increased epinephrine and norepinephrine levels, potentially explaining the efficacy of beta-blockers in this setting. However, careful monitoring is necessary, as beta-blockers can also lead to conduction disorders in transposition of the great arteries.

As cardiac fibrosis is a common pathway leading to myocardial dysfunction, aldosterone blockage could be useful as it is thought to prevent myocardial fibrosis [14]. Eplerenone, an aldosterone blocker, has been assessed in small cohorts of patients (27 patients in sum) [15,16] and failed to prove any significant effect on RV mass changes, exercise capacity, serologic markers of collagen turnover, and inflammation.

Angiotensin II receptor blockers (ARBs) such as valsartan significantly reduce morbi-mortality in HFREF [17]. Several studies attempted to assess the effect of ARBs in sRV. First, losartan has failed to improve exercise capacity or natriuretic peptides [18]. In a randomized trial, there was no significant effect of valsartan on RV ejection fraction, exercise capacity, or quality of life [19]. Ramipril, an angiotensin converting enzyme (ACE) inhibitor, in a small but prospective, randomized controlled clinical trial, has also failed to improve RV function in adult patients with sRV after Senning or Mustard procedure [20]. Thus, until today, there is no conclusive evidence regarding the beneficial effect of ACE inhibitors or ARBs in adult patients with systemic RVs. Data from prospective controlled studies are scarce and valuable but can be considered as underpowered. Based on HF prevalence in patients with an sRV who did not receive ARBs after a median follow-up of 8.3 years in van Dissel et al. study [21], we estimated that 199 patients would be required to be enrolled in each study group to achieve 80% power at the 5% significance level (two-sided) to detect an absolute reduction of 10% over a 7-year follow-up (i.e., 20% in the control group and 10% in the treated group) [22]. Such a design appears unrealistic. However, using a different approach, we demonstrated in a retrospective observational cohort including 359 patients with an sRV the lack of significant association between renin–angiotensin–aldosterone system inhibitors and a reduced HF incidence or mortality [22].

Angiotensin receptor–neprilysin inhibitors (ARNI) have emerged as a standard of therapy for adults with HF [23]. Conflicting data have been presented about ARNIs in ACHD patients. Data from case reports and prospective cohorts indicate improvements in NT-pro-BNP and sRV function [24,25,26,27]. Overall, ARNIs seem to be very well tolerated in patients with a sRV [25]. However, in a small retrospective cohort including 12 patients with a sRV [28], sacubitril/valsartan failed to improve sRV function and exercise capacity. More recently, sacubitril/valsartan has been well tolerated and associated with improved sRV function, clinical status, and exercise capacity in two larger cohorts of patients [26,27].

Finally, tadalafil, a phosphodiesterase-5 inhibitor, has been studied in the SERVE trial [29], but results have not yet been published.

### 2.4. Failing Fontan

The Fontan procedure is one of the great advances of CHD surgery that has been successful in treating patients with single ventricle physiology by eliminating cyanosis and chronic volume overload. The Fontan circulation creates a direct connection between the systemic veins and the pulmonary arteries, bypassing the heart and allowing for direct flow into the pulmonary arteries without the use of a ventricular pump. However, many patients who undergo Fontan-type operations experience complications, such as reduced exercise tolerance, leg edema, pleural effusions, ascites, liver disease, arrhythmia, and protein-losing enteropathy (PLE), which can negatively impact their quality of life and increase their mortality [30]. To optimize the Fontan circulation, it is crucial to maintain low pulmonary vasculature resistance (PVR) and preserve both the systolic and diastolic function of the single systemic ventricle. However, a “failing Fontan” circulation is an almost inevitable long-term consequence of the altered physiology apparent in an increasing number of patients, and despite efforts to correct structural lesions using interventional procedures, increased PVR and progressive single ventricle dysfunction remain important contributors to late circulatory failure [31].

For the past decade, pulmonary vascular disease has been suspected to be the primary mechanism for late Fontan failure. A study based on autopsies described a unique remodeling of the pulmonary arteries with intimal hyperplasia and media regression in patients with late failing Fontan, which has been attributed to long-term non-pulsatile low-shear flow of the Fontan circulation [32,33]. Consequently, most randomized control trials assessing drug effects in Fontan patients have targeted PVR with limited and controversial outcomes. A meta-analysis including nine studies examining therapy for pulmonary arterial hypertension in Fontan patients showed significant improvements in hemodynamics, functional class, and 6 min walk distance [34]. However, there were no changes observed in mortality or levels of N-terminal proB-type natriuretic peptide (NT-proBNP) [34]. Most of these studies had a short duration, small sample size, and mainly included stable and asymptomatic Fontan patients. Two randomized clinical trials have confirmed that vasodilators have a limited effect on Fontan patients. The TEMPO study, which involved 69 patients, reported an improvement in exercise capacity with the use of bosentan, resulting in a modest increase in exercise duration [35]. In the Fontan Udenafil Exercise Longitudinal (FUEL) multicenter trial involving treatment with udenafil in 400 asymptomatic Fontan patients, no improvements were observed in oxygen consumption at peak exercise, NT-ProBNP level, or myocardial performance index [36]. Finally, the Prospective, Multi-center, Double-blind, Randomized, Placebo-controlled, Parallel-group Study evaluating the Efficacy and Safety of Macitentan in Fontan-palliated Adult and Adolescent Subjects (NCT03153137) trial, which compared the efficacy and safety of macitentan with a placebo in asymptomatic Fontan patients, was terminated prematurely due to a lack of significant clinical benefits (results are not yet published). None of these trials showed a decrease in late failing Fontan incidence. Two studies described PAH therapies used in failing Fontan. The first was a multicentric observational study that found functional class was more likely to improve in treated patients after a median of 12 months [37]. The second was an open label, non-controlled study that failed to show an overall significant improvement after 3 months of treatment with bosentan in 10 patients with a failing Fontan although saturations of oxygen and/or exercise capacity improved in half [38]. These controversial results may be explained by an inadequate choice of therapeutic target knowing that multiple mechanisms are at the origin of Fontan failure, and that the increase in PVR is involved in less than 20% of Fontan failures [31]. Consequently, European guidelines recommend considering pulmonary arterial hypertension therapy in selected Fontan patients with elevated pulmonary resistance (PVR > 2 indexed Wood units) in the absence of high ventricular end diastolic pressure [39].

A recent study with longitudinal hemodynamic monitoring demonstrated that systolic (46%) but also diastolic (31%) single ventricle dysfunction were the most prevalent etiologies of late Fontan circulation failure [31]. The systemic single ventricle is subjected to a series of stresses, ranging from extreme volume overload to decreased preload after Fontan completion. These stresses can lead to the development of eccentric hypertrophy, dilation, as well as systolic and diastolic dysfunction. Despite this, there is currently no standardized management plan for treating or preventing systolic and diastolic dysfunction in Fontan patients. Furthermore, there is no evidence that HF therapy, which is commonly used for patients with acquired heart disease, can effectively alleviate symptoms or improve the prognosis of Fontan patients, notably when the Fontan circulation is failing. In one randomized study of angiotensin-converting enzyme inhibitors (enalapril) in Fontan patients without congestive HF, the active treatment group showed lower cardiac index during exercise [40]. Preload deprivation represents the Achilles’ heel of Fontan physiology and can result from such treatments in asymptomatic patients. A recent crossover trial found that carvedilol did not improve exercise performance and was associated with a mildly increased N-terminal-proBNP level [41]. Finally, it is unknown whether the significant benefits of sacubitril/valsartan and SGLT2i in HF in the absence of CHD would have a similar impact on diuresis and prevention of adverse cardiac remodeling in patients with failing Fontan.

The different trials of HF treatments evaluated in ACHD are shown in Table 1, according to heart failure pathophysiology.

## 3. Anticoagulation Therapy in ACHD

### 3.1. Antithrombotic Therapy in ACHD

Thromboembolism (TE) is a significant risk factor for ACHD, accounting for 4% of the overall CHD mortality [52,53]. Notably, the incidence of stroke is between 0.05 to 0.8% per year and may lead to neurological sequalae [53,54,55]. This risk is higher than in the general population especially in younger age and complex CHD [55,56]. The incidence of venous thrombosis and pulmonary embolism is also dependent on the CHD but remains less studied.

Anticoagulation prophylaxis is commonly recommended in ACHD patients with an increased risk of thromboembolic events, such as those with cyanotic heart disease, Fontan circulation, mechanical heart valves, or atrial arrhythmia. However, given the heterogeneity of ACHD patients and risk stratification, curative and preventive thromboembolic treatment remains challenging [55]. Indeed, randomized clinical trials to guide anticoagulation strategies are very scarce resulting in guidelines that predominantly rely on level C of evidence [39,57]. Despite the lack of prospective studies in ACHD, non-vitamin K antagonist oral anticoagulants (NOACs) are increasingly replacing VKAs and now account for almost half of all oral anticoagulant prescriptions [58]. The absence of monitoring and dose adjustments with NOAC appears particularly attractive for the young and active ACHD patients. However, the lack of high-quality evidence to guide the optimal duration and intensity of anticoagulation prophylaxis in ACHD emphasizes the need for further research in this area, as it is a commonly used treatment modality [55].

### 3.2. Fontan Circulation

TE event is a common complication after Fontan surgery and is one of the main causes of long-term mortality [59]. The risk of thrombosis is increased by abnormalities of coagulation, platelet activation, and endothelial dysfunction leading to hypercoagulability [55]. Therefore, risk stratification and the choice of therapeutic drugs for preventing thromboembolic events remain topics of debate and exhibit significant variability across different centers. American [57] and European guidelines [39] do not recommend the use of NOAC because robust data are lacking. Very recently, a meta-analysis including 21 studies compared the efficacy and safety of aspirin, warfarin, and NOAC in Fontan circulation [60]. Only three of the included studies reported outcomes of NOACs use in Fontan patients, which accounted for only 1.2% of the total patient-years. This meta-analysis, as well as another one, confirms that any prophylaxis decreases the risk of TE event compared with no prophylaxis [60,61]. Interestingly, NOACs were associated with the strongest reduction in TE events, but also had the greatest risk of major bleeding. Notably, the risk of uterine bleeding should be considered when administering NOACs to this population. Furthermore, the optimal dose of NOAC in primary prophylaxis has to be defined. Overall, the conclusion of the meta-analysis supports the safety and efficacy of NOAC in the treatment of patients with a Fontan circulation despite the limited number of patients and the heterogeneity of studies using NOAC. On the other hand, aspirin tended to have the most favorable overall profile, with both a significantly reduced risk of TE events and a tendency to lower risk of major bleeding [60,61]. However, more than 50% of aspirin-treated patients have aspirin resistance shown by ex vivo dynamic thromboxane B2 generation evaluation. This measurement may help account for the ineffectiveness of aspirin in preventing thrombotic complications in some patients [62]. Nevertheless, VKA or NOAC have to be preferred to aspirin in atrio-pulmonary connection, atrial arrhythmia, or previous history of TE.

### 3.3. Eisenmenger Syndrome

In patients with and without CHD, pulmonary hypertension is associated with an increased risk of thrombosis, especially in pulmonary arteries [63]. Patients with Eisenmenger syndrome exhibit a hypercoagulability state due to erythrocytosis secondary to hypoxemia, shear stress leading to endothelial dysfunction, and platelet over aggregation [64,65]. On the opposite end, patients also exhibit a hypocoagulability state due to impaired fibrinogen function, higher hematocrit and platelet activity contributing to the risk of hemoptysis [66,67].

Anticoagulation is used in almost half of patients without CHD and pulmonary arterial hypertension, but no robust data allow recommendations, so individual decision-making is required [8]. In patients with pulmonary hypertension and CHD, the decision to use oral anticoagulation should be made through a case-by-case evaluation, carefully balancing the risk of thromboembolism against the risk of hemorrhage, especially when concomitant TE risk factors exist such as atrial arrhythmias, HF but in absence of hemoptysis [8]. No specific data has been published about the efficacity and safety of NOAC in Eisenmenger syndrome patients. Nevertheless, in the nationwide German study on anticoagulation use in ACHD, 22% of patients with Eisenmenger syndrome were anticoagulated, of which half with NOAC, but no analysis of this subgroup were available [58]. An individual approach is needed to determine the most appropriate thromboprophylaxis therapy.

### 3.4. Atrial Arrythmia

For patients with atrial arrhythmia and simple CHD, anticoagulation is guided by the CHA2DS2-VASc score; whereas in those with moderate to complex CHD, anticoagulation is recommended regardless of the score. NOACs seem similarly safe and effective in the general population [39]. Data from the prospective NOTE registry encouraged their use as they showed short-term safety and efficacy of NOAC in 530 ACHD patients mainly with atrial arrythmia and moderate or severe CHD [68].

### 3.5. Antiplatelet Agents after Device Implantation

Antithrombotic therapy following transcatheter ASD closure remains empirical, with aspirin for 6 months being commonly prescribed. Two RCT demonstrated the addition of clopidogrel to aspirin in patients who underwent transcatheter ASD closure was associated with a reduced monthly frequency of migraine attacks over 3 months, compared to aspirin alone [69,70]. Aspirin therapy was also administered for at least one year after implantation of balloon-expandable stents for coarctation of the aorta, and no patient showed evidence of thromboembolism after an average follow-up period of 18 months [71]. Antiplatelet agents are typically prescribed empirically for 3 to 6 months after surgically or percutaneously transpulmonary pulmonary valve replacement. It is noteworthy that current guidelines, including those related to valvular and congenital heart conditions, do not address medical therapy after pulmonic bio prosthesis replacement. However, no benefit has been observed from long-term medication (>6 months) after pulmonary valve replacement using a bioprosthetic valve [72].

### 3.6. Non-Vitamin K Antagonist Oral Anticoagulants

The increased use of NOACs in real-world over the last few years in the treatment of patients with CHD was observed in the nationwide study from one of Germany’s largest health insurers, which included 44,097 ACHD patients on oral anticoagulation [58]. Between 2005 and 2018, the use of oral anticoagulants in ACHD increased from 6.3% to 12.4%, and the utilization of NOACs accounted for 45% of prescribed anticoagulants in ACHD in 2018. Results were striking since patients treated with NOACs were associated with excess long-term risk of major adverse cardiovascular events and all-cause mortality but also bleeding when compared to outcomes under VKAs. However, findings from the initial international multicenter prospective cohort study from NOTE registry indicated that NOACs could be a safe and potentially effective option for preventing thromboembolic events in ACHD [68].

The difference can be partly explained by the differences in the population study and the endpoints definition. In the German cohort, patients were older (69 versus 47 years old) with mainly simple CHD (64% versus 15%) and higher CHA2DS2-VASc score. Thus the 1-year rate of major TE events was significantly higher (3% versus 1%), and the 1-year rate of major adverse events (a composite of acute myocardial infarction, ischemic stroke, ventricular flutter/fibrillation, resuscitation, or death) reached 6.8% in the Freisinger et al. study. The authors point out that most of the patients were likely community based, since up to half of patients with ACHD in Germany had no specialized ACHD follow-up. Therefore, these therapies should be carefully prescribed and supervised by ACHD cardiologists.

## 4. Antiarrhythmics Treatments

Supraventricular tachycardias (SVT) are a significant contributor to morbidity and mortality in individuals with CHD. However, treating arrhythmias in individuals with CHD is challenging due to the altered anatomy and physiology of the heart. While evidence-based guidelines for pharmacologic treatment of atrial arrhythmias exist for the general adult population, there is a dearth of data on the efficacy and safety of these treatments in the adult CHD population. The priority in all CHD patients is to maintain sinus rhythm. Catheter ablation is the preferred first-line treatment over long-term pharmacological therapy since antiarrhythmic drugs often have negative inotropic and/or dromotropic effects and a low efficacy at mid-long-term [73]. A multicentric retrospective study reported that only 45% of treated patients remained free from SVT after 2.5 years of follow-up under antiarrhythmics [74]. Class III antiarrhythmics (85% sotalol and 15% amiodarone) were found to be significantly more effective in reducing the risk of SVT recurrence. However, adverse medication effects occurred in 22% of the patients, predominantly in those taking amiodarone. These results were also observed with low-dose amiodarone (<200 mg/day), with most side effects consisting of thyroid dysfunction [75]. Because sotalol is associated with fewer adverse effects than amiodarone, it should be considered as the first-choice therapy. Amiodarone remains an option in CHD patients with systemic ventricular dysfunction.

Ventricular arrhythmia-related sudden cardiac death is a significant concern, accounting for up to 25% of all ACHD deaths [76]. Antiarrhythmic drugs may be used as an adjunct to defibrillator (ICD) to reduce ICD therapies. There is a lack of specific studies regarding the effectiveness of antiarrhythmic drugs in reducing ICD appropriate shocks in ACHD. Historical case series suggest that mexiletine [77] and phenytoin [78] can reasonably suppress ventricular arrhythmias, while sotalol and amiodarone may also be effective [75,79]. Dofetilide may be an acceptable alternative to amiodarone when there is ventricular dysfunction, assuming there is no impaired renal function or prolonged QTc interval.

## 5. Cardiac Drugs in CHD-Related Pulmonary Arterial Hypertension

PAH is an uncommon but life-threatening complication of CHD, particularly in its most advanced form, the Eisenmenger syndrome (ES). The incidence of PAH related to CHD varies worldwide, but it is estimated up to 10% [80,81].

Advances in therapeutic strategy have improved life expectancy of PAH-CHD patients in recent decades [80]. Nevertheless, mortality continues to be high, especially in ES, with a 5-year survival estimated between 74% and 81% [82,83]. Over the last few decades, there has been a shift in the specific causes of mortality in ES. Mortality due to HF (HF) appears to be predominant (34%) in contrast to mortality due to hemorrhagic events (7%) and peri-procedural deaths (7%) [82]. This may be partly explained by the increased longevity of ES patients in the current era of advanced therapy. These patients will die later and from chronic rather than acute cardiac causes. To establish the therapeutic strategy, the singularity and particularities of each patient, the CHD type, the type of surgical or interventional correction, the functional state, and the hemodynamic data are considered.

Currently available PAH-specific drug therapies (PAH-SDTs) target pulmonary arterial endothelial dysfunction, primarily aiming to induce vasodilation and interfere with the remodeling of the pulmonary microvascular tree [84].

PAH-SDTs have reported to improve outcomes in ES [80,85,86]. However, current evidence supporting PAH-SDTs in ES remains limited and based mainly on expert opinion. Additionally, current risk scores for escalating treatment and timing of transplantation cannot be extrapolated to ES patients [87]. Most of the larger randomized clinical trials (RCT) have been performed on heterogeneous PAH populations with a small percentage of ES patients.

Bosentan, a dual endothelin receptor antagonist (ERA), was the first drug to demonstrate in an RCT (BREATHE-5) an improvement in hemodynamics (reduction of pulmonary vascular resistance) and exercise capacity (increase in 6 min walking distance) in patients with New York Heart Association/World Health Organization functional class (NYHA/WHO FC) III ES [88]. Bosentan is recommended in symptomatic patients (NYHA/WHO FC ≥ II) to improve exercise capacity (ESC/ERS PAH guidelines 2022, Class I, level of evidence B) [8]. Ambrisentan is a selective mono-antagonist of the ET-A endothelin receptor with a longer half-life than bosentan, allowing it to be given once daily. A retrospective study showed an improvement in T6M in patients with PAH-CHD [80]. In the MAESTRO RCT study, macitentan, new-generation ERA, found no effect on 6MWD in a mixed cohort of patients with ES, although a decrease in NT-proBNP and PVR was observed [89]. This study enrolled a more heterogeneous population compared with BREATHE-5, including patients with complex cardiac defects, NYHA/WHO FC II, and Down syndrome. Smaller RCTs and prospective trials on phosphodiesterase type 5 (PDE-5] inhibitors also have shown favorable hemodynamic and functional effects in ES [90,91,92,93].

Scarce data are available on prostanoids in symptomatic ES, mainly used as a third line therapy [94,95,96,97]. Retrospective studies observed that continuous intravenous prostacyclin analogs (epoprostenol and treprostinil) in symptomatic patients with ES improved hemodynamics and functional capacity [94,98]. In a small, single-center pilot study, adding nebulized iloprost to oral PAH-SDT failed to improve 6 MWD in ES [97].

New generation PAH-SDT, such as riociguat (a soluble guanylate cyclase stimulator) and selexipag (a new oral selective IP prostacyclin receptor agonist), have demonstrated functional and hemodynamic improvement in patients with PAH post shunt repair [99,100,101].

PAH-SDT is based on a stepwise intensification therapy [8]. However, experience with sequential combination therapy in patients with PAH-CHD is limited and challenging.

An RCT analyzing the combination therapy with bosentan and sildenafil in ES patients demonstrated an increase in SaO2 when sildenafil was added but without additional benefits on hemodynamics or exercise capacity [102]. Another prospective study showed better functional capacity and hemodynamic response with combination therapy (bosentan and sildenafil) in ES patients who deteriorated clinically on monotherapy [103].

Triple combination therapy in ES requires further investigation. Only one retrospective cohort study observed clinical and hemodynamic benefits with parenteral prostanoid in ES patients on dual oral combination therapy [104].

Furthermore, PAH-SDT strategy might differ according to the type of lesion and its location. Patients with small/coincidental defects and patients with PAH after defect correction have a physiopathology close to those of patients with idiopathic PAH and should be treated following the current guidelines for these patients. A combination therapy strategy (ERA + PDE-5 inhibitor) in patients at low and intermediate risk, and the addition of parenteral prostanoids in high-risk patients as in idiopathic PAH [8], could be considered.

Patients with complex lesions and post-tricuspid lesions seem to be less likely to respond to PAH therapies compared with patients with simple lesions [86]. Sequential combination therapy should be considered in patients with PAH-CHD, including ES, if they do not meet treatment goals (ESC/ERS PAH guidelines 2022, Class IIa, level of evidence C) [8].

Treatment options for HF in PAH-CHD are limited. Right-sided HF is predominant in ES patients, and its treatment will be mainly symptomatic with the use of diuretics (loop diuretics, thiazides, and mineralocorticoid receptor antagonists used as monotherapy or in combination) preventing systemic fluid retention [8]. No data from RCT are available on the usefulness and safety of specific left-side HF therapies (β-blockers, angiotensin-converting enzyme-inhibitors, angiotensin receptor-neprilysin, sodium-glucose cotransporter-2 inhibitors) in PAH [8,82,105]. A prospective study suggested that b-blockers therapy (metoprolol) may improve effort tolerance in selected ES patients [8,106]. Left-side HF therapies are not recommended in patients with PAH unless it is required by additional comorbidities (i.e., high blood pressure, coronary artery disease, associated left-side HF, or arrhythmia). They should be used cautiously and only under expert judgement. Adverse effects, such as peripheral vasodilation and bradycardia, might have a detrimental benefit in PAH-CHD patients. Moreover, iron deficiency is common in patients with PAH, and it is associated with impaired myocardial function, aggravated symptoms, and increased mortality risk. Supplementation iron treatment should be considered in patients with iron deficiency (ESC/ERS PAH guidelines 2022, Class IIa, level of evidence C) [8].

Finally, risk stratification methods specific to ES are lacking. Risk stratification is essential to predict survival, define treatment goals, and guide management (treatment escalation and timing for transplantation). Specific risk stratification scores for PAH-CHD population are thus needed based on existing multi-parametric models [107].

## 6. Conclusions

In conclusion, the management of cardiovascular disease ACHD continues to be challenging. The use of cardiac drugs in this population is based on the rationale of their efficacy in the general population, but their application to ACHD patients is limited by a lack of evidence. There is a growing body of literature supporting the use of certain drugs, such as HF drugs or NOACs, but much of the evidence is from small and/or retrospective studies. Even in PAH therapies, risk stratification to guide sequential or combination therapy and to appropriately refer the patient for (heart and) lung transplantation is lacking. There are knowledge gaps in the optimal dosing and the potential interactions between ACHD-specific comorbidities or treatments and cardiac drugs. Future research is needed to better define the role of cardiac drugs in the management of ACHD cardiovascular disease, with a focus on larger, prospective studies and more detailed analysis of the unique aspects of ACHD physiology and treatment, keeping in mind the ambitious aims to reduce long-term cardiovascular complications and improve quality of life of these patients.

## Figures and Tables

**Table 1:** Main trials on cardiac drugs in ACHD-related heart failure.

Study Design	Subaortic LV Failure	Sub-Pulmonary RV Failure	Subaortic RV Failure	Failing Fontan
RCT or Meta-analysis	Ramipril in ToF (post-hoc analysis of APPROPRIATE study): stabilization of LV and RV function [7]	Losartan in ToF (REDEFINE): no effect on RVEF [9] PAH therapies (Section 3)	Eplerenone: no effect on sRV mass and EF, neurohormonal and collagen turnover biomarkers [15] Losartan [18], valsartan [19]: no effect on sRVEF, exercise capacity, and NT-proBNP Ramipril: no changes in sRV volumes and EF [20] Tadalafil (SERVE): no change in sRV volume or sRVEF (results communicated at ESC congress 2022, not yet published) SGLT2i (ongoing studies: NCT05580510)	Bosentan: conflictual effect on peakVO2 and NYHA, no effect on NTproBNP and QoL [35,42] Ambrisentan: improvement of peak VO2 [43] Macitentan (not yet published) Udenafil (FUEL): no improvement in peak VO2 [36] Sildenafil: conflictual results on peak VO2 [44,45]; no change in pressure-volume loop [46]; increase in cardiac index [47] Iloprost: improvement of peak VO2 [48] Meta-analysis [34]: significant improvements in hemodynamics, functional class, and 6 min walk distance but no changes in mortality or NT-proBNP Enalapril in asymptomatic patients: decrease in cardiac index [40] Carvedilol: no change in exercise performance and mild increase in NT-proBNP level [41]
Open label trial	No data	No data	Eplerenone: no effect on collagen biomarkers, 6MWD, or QoL [16]	Bosentan: no changes in saturations of oxygen, exercise performance, and QoL [38]; improvement of 6MWD and MRI-derived resting cardiac output [49] Sildenafil: improvement of peak VO2 [50]
Prospective observational studies	Guideline-directed medical therapy would improve LVEF [3]		Β-blockers may improve NYHA, QoL, +/− sRVEF [12] ACEi or ARBs: no association with a reduced HF incidence or mortality [22] ARNI: conflicting data but seem to be associated with improvement of NT-proBNP level, sRV function, NYHA, 6MWD, and QoL [24,25,26,27,28] SGLT2 i: one clinical case with functional and echocardiographic improvement [51]	PAH therapy: improvement of NYHA functional class in patients with PAH therapies [37]
Recommendations	Diuretics, ACE inhibitors, ARBs, angiotensin receptor/neprilysin inhibitors, mineralocorticoid receptor antagonists, βblockers, and SGLT2i should be used	Losartan should not be prescribed routinely in ToF to prevent the progression of RV dysfunction and RV HF	No evidence for eplerenone, ACEi, ARBs, βblockers, sacubitril/valsartan effects in sRV Valsartan or sacubitril/valsartan should be considered in symptomatic patients No data yet on SGLT2i use	Considering PAH therapy in selected Fontan patients with elevated PVR (>2 indexed Wood units) in the absence of high ventricular end diastolic pressure Avoiding βblockers and ACEi specifically in patients without systolic ventricular dysfunction and increased end-diastolic ventricular pressure No data yet on ARNI and SGLT2i use

6MWD, 6 min walk distance; ACE, angiotensin converting enzyme; ARBs, angiotensin II receptor blockers; ARNI, angiotensin receptor-neprilysin inhibitor; EF, ejection fraction; HF, HF; LV, left ventricle; NT-proBNP, N-terminal pro b-type natriuretic peptide; NYHA, New York Heart Association; PAH, pulmonary arterial hypertension; PVR, pulmonary vascular resistance; QoL, quality of life; RCT, randomized clinical trial; RV, right ventricle; SGLTi, sodium-glucose cotransporter 2 inhibitor; sRV, systemic right ventricle; ToF, tetralogy of Fallot.

## Data Availability

No data was generated for this review.
